# Modulation of Photosensitizing Responses in Cell Culture Environments by Different Medium Components

**DOI:** 10.3390/ijms251810016

**Published:** 2024-09-17

**Authors:** Hyowon Lee, Jungil Hong

**Affiliations:** Department of Food Science and Technology, College of Science and Convergence Technology, Seoul Women’s University, Hwarang-ro 621, Nowon-gu, Seoul 01797, Republic of Korea; hyowon24@korea.ac.kr

**Keywords:** culture media, riboflavin, phenol red, light irradiation, HaCaT phototoxicity, serum

## Abstract

Many cell culture experiments are performed under light to evaluate the photodynamic or photosensitizing efficacy of various agents. In this study, the modulation of photosensitizing responses and phototoxicity under cell culture conditions by different medium components was investigated. The significant levels of reactive oxygen species (ROS) generated from DMEM, RPMI 1640, and MEM were observed under the irradiation of fluorescent light (FL) and white and blue LEDs, indicating that these media have their own photosensitizing properties; DMEM showed the most potent property. Phenol red-free DMEM (Pf-D) exhibited a stronger photosensitizing property than normal DMEM by 1.31 and 1.25 times under FL and blue LEDs, respectively; phenol red and riboflavin-free DMEM (PRbf-D) did not show any photosensitizing properties. The inhibitory effect on light transmission was more pronounced in DMEM than in RPMI, and the interference effect on green LED light was greatest at 57.8 and 27.4%, respectively; the effect disappeared in Pf-D. The media containing riboflavin induced strong phototoxicity in HaCaT keratinocytes by generating H_2_O_2_ under light irradiation, which was quenched by sodium pyruvate in the media. The presence of serum in the media was also reduced the phototoxicity; H_2_O_2_ levels in the media decreased serum content dependently. The phototoxicity of erythrosine B and protoporphyrin IX under FL was more sensitively pronounced in PRbf-D than in DMEM. The present results indicate that several medium components, including riboflavin, phenol red, sodium pyruvate, and serum, could modulate photosensitizing responses in a cell culture system by inducing photosensitizing activation and by interfering with irradiation efficacy and ROS generation.

## 1. Introduction

A variety of animal models have been used in biomedical research because they are important experimental subjects due to their physiological similarity to humans. Animal studies have also been used extensively to evaluate the safety and efficacy of new drugs, as well as to assess various toxicities [1]. However, growing ethical concern regarding animal suffering and the morality of experimentation have led researchers to explore alternative methods, such as computer modeling, organoids, and cell or tissue culture [2,3].

Cell culture-based studies allow researchers to control the experimental environment with high precision, including temperature, pH, oxygen levels, osmotic pressure, and nutrient availability. This control can lead to more consistent and reproducible results [4]. In addition, recent advances in co-culture systems and 3D culture models have improved the accuracy of cell experiments by more effectively simulating the biological environment with an actual living organism in vivo [5,6]. The advancement of these cellular models is critical in areas such as disease modeling, drug development, and regenerative medicine [7,8].

In cell experiments, culture media are essential for cells to grow, divide, and perform their physiological functions in vitro and vary widely in composition and concentration. These media typically contain nutrients such as *D*-glucose, amino acids, and vitamins, as well as basal salts and buffering agents to adjust pH and osmotic conditions, and phenol red, the pH indicator. Among these components, riboflavin, a member of the B group of vitamins, is essential for cell growth and serves as a precursor to biologically active cofactors including flavin mononucleotide (FMN) and flavin adenine dinucleotide (FAD). Despite its widely known photosensitizing properties, the effects of light irradiation on riboflavin-containing media have not been well studied [9]. Furthermore, while various applications of light, such as photodynamic therapy, have been extensively studied using experimental cell models, potential sources of interference in these experiments, such as photosensitizers, light-interfering dyes, and serum in the media used during light irradiation, have not been thoroughly investigated.

Since the skin is a critical barrier against various light sources, including sunlight, skin photoaging, including both acute and chronic damage caused by UV exposure, has been actively studied. More recently, studies have begun to explore the effects of visible light on the skin, including erythema and hyperpigmentation [10]. Visible light of various wavelengths is also used to treat conditions such as psoriasis, hair regeneration, wound healing, tattoo removal, and injury treatment [11,12,13,14,15]. Another therapeutic approach to cancer is photodynamic therapy, which uses photosensitizers to induce biological effects [16]. The increasing incidence of skin-related diseases and the growing interest in skin health have spurred growth in the development of functional cosmetics [17]. Since 2013, however, the European Union (EU) has banned the production and sale of cosmetics tested on animals. As animal testing bans for cosmetics have spread worldwide, in vitro testing has expanded, underscoring the growing importance of cell-based assays [18,19]. However, the effects of experimental conditions in the cell system on light transmission and the induction of photosensitizing properties, and the resulting effects, are often not fully considered in related experiments.

Despite the essential role of cell culture media in experimental contexts, the impact of media in light-based experiments remains underexplored. Therefore, this study aims to investigate the effects of cell culture media under light irradiation conditions on photosensitizing responses. In this study, media commonly used in cell culture experiments were examined for the expression of their own photosensitizing properties and the consequent effects. In addition, the influence of riboflavin and other components on the expression of the photosensitizing property of cell culture media and on the growth of HaCaT, or human keratinocytes, was investigated. The aim of this study was to provide fundamental insights into the factors and confounding effects that should be considered when conducting cell experiments involving light irradiation.

## 2. Results and Discussion

### 2.1. Evaluation of Photosensitizing Properties of Cell Culture Media under Different Lights

With the development of various biomedical applications utilizing different types of light, the use of light in cell culture systems has become an increasingly active area of research. Light-emitting diodes (LEDs), which consist of P- and N-type semiconductors, efficiently convert electrical energy into light. LEDs are widely employed in digital displays, general lighting, and biomedical applications due to their high energy efficiency and long lifespan [20]. In particular, research has demonstrated that blue light irradiation can induce apoptosis in *Propionibacterium acnes*, leading to beneficial effects on skin health. Sequentially pulsed LED light at 660 nm has also been shown to improve skin appearance by up-regulating collagen production and down-regulating matrix metalloproteinase-1 [21,22]. In addition, the combination of low-level red and near-infrared LED light has been reported to further improve skin appearance by increasing collagen and elastin production [23]. The application of LEDs is expanding in various fields, including cosmetics, medicine, and antibacterial treatments, with numerous studies reporting on the effects of LED light in cell experiments. With the increasing use of LEDs in these research areas, it is critical to confirm how interactions between cell culture media and light, including different LEDs, might influence experimental outcomes.

In this study, three commonly used culture media—Roswell Park Memorial Institute 1640 (RPMI), Eagle’s Minimum Essential Medium (MEM), and Dulbecco’s Modified Eagle’s Medium (DMEM)—as well as phenol red-free DMEM (Pf-D) and phenol red- and riboflavin-free DMEM (PRbf-D), were exposed to various light sources. The levels of reactive oxygen species (ROS) generated through the photosensitization of the media were evaluated using a 2′,7′-dichlorofluorescin (DCFH) probe.

**Figure 1:**
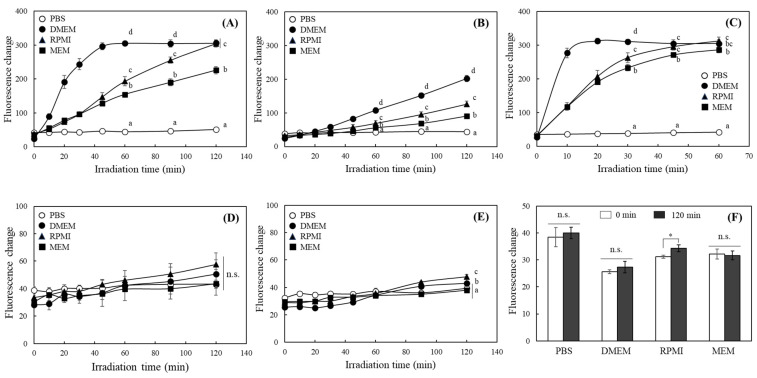
Evaluation of photosensitizing properties of different media under various lights. PBS and different media, namely DMEM, RPMI, and MEM, were incubated under the irradiation of fluorescent light (FL, 2000 lx) (**A**), white (2000 lx) (**B**), blue (200 lx) (**C**), red (2000 lx) (**D**), and green (2000 lx) (**E**) LEDs, and in the dark (**F**). Changes in the fluorescent intensity of DCFH were analyzed (ex. 485, em. 535 nm) at each time point. Each value represents the mean ± SD (*n* = 3). Different letters indicate a significant difference (*p* < 0.05) based on one-way ANOVA and Tukey’s HSD test (in (**A**–**E**)). Significantly different from each other based on Student’s *t*-test (*, *p* < 0.05 in (**F**)). n.s.; not significant.

First, the photosensitizing property of commonly used media containing phenol red and phosphate-buffered saline (PBS) was measured under the irradiation of FL; red, green, and white LEDs (each 2000 lx); and 200 lx blue LEDs (Figure 1). The results show the significantly different responses of 2′,7′-dichlorofluorescein (DCF) fluorescence among the media under the irradiation of different lights. DMEM showed the fastest increase and strongest intensity of DCF fluorescence, followed by RPMI under FL irradiation; no significant changes in the fluorescence were observed in PBS (Figure 1A). Similar results were observed in the media exposed to white and blue LEDs, with the greatest increase in DCF fluorescence under blue LED irradiation (Figure 1B,C). However, a slight increase in DCF fluorescence was observed in the media exposed to red and green LEDs (Figure 1D,E); no significant differences in fluorescence over time in the dark were observed (Figure 1F).

**Figure A1:**
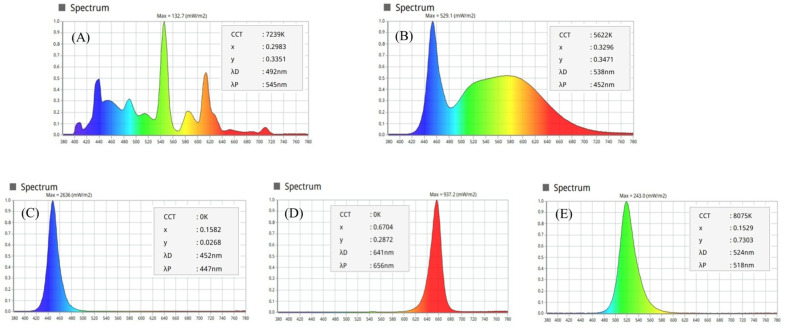
Emission spectra and characteristics of the irradiated lights including regular fluorescent light (**A**) and white (**B**), blue (**C**), red (**D**) and green (**E**) LEDs used in the present study. Different colors in subfigures represent intrinsic visible light colors with peaks at those wavelengths. CCT, color correction temperature; x and y, CIE1931 color coordinate; λD, dominate wavelength; λP, peak emission wavelength.

The increase in DCF fluorescence under the three light sources, including FL and white and blue LEDs, was the same in the order of DMEM, RPMI, and MEM. The riboflavin content seems to play the most important role in the expression of the photosensitizing properties of these media, considering that DMEM, RPMI, and MEM contain 0.4, 0.2, and 0.1 mg/L of riboflavin, respectively (Table A1). Riboflavin is a well-known photosensitizer, absorbing light in the 440–450 nm blue light range [24]. In this study, each medium exhibited the greatest photosensitizing property under blue LEDs, with a peak emission wavelength of 447 nm, and barely increased DCF fluorescence under red or green LEDs with peak emission wavelengths of 656 and 518 nm, respectively, suggesting that riboflavin plays a critical role in photosensitizing activity (Figure 1D,E). FL and white LEDs, on the other hand, have a wide range of emitting wavelengths capable of inducing photosensitization of riboflavin (Figure A1), which is thought to cause photoreactivation of media.

### 2.2. Evaluation of the Interference Effect of Culture Media on Light Transmission

Phenol red is a pH indicator commonly added to most cell culture media. It changes color depending on the pH of the medium; phenol red gives the medium a red or pink color at a neutral pH (~7.4). The concentration of phenol red in typical cell culture media is 5–15 mg/L [25,26]. Although this concentration of phenol red does not significantly affect the biological processes in most cell culture applications, it provides an obvious difference in color of medium for pH indication as a dye component. Therefore, the color of phenol red is expected to be sufficient to interfere with light transmittance in light-applied cell experiments.

**Figure 2:**
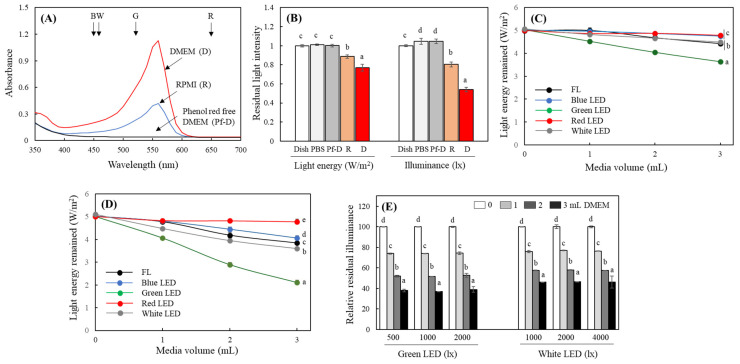
Effects of different culture media on light transmittance. Absorbance spectra of different media were analyzed (**A**). Changes in light energy and illuminance after transmission of FL (5 W/m^2^ and 2000 lx) through culture dish alone (35 mm × 10 mm), or the dish containing PBS, DMEM (**D**), phenol red-free DMEM (Pf-D), and RPMI (R) medium (each 3 mL) were analyzed (**B**). Changes in the interference effect of RPMI (**C**) and DMEM (**D**) by different medium volume (0–3 mL) on different light sources (each 5 W/m^2^) were measured. Effects of DMEM (3 mL) on transmittance of green and white LEDs at different illuminance were also analyzed (**E**). Each arrow indicates the emission peak of blue (B), white (W), green (G), and red (R) LEDs used in this study (in (**A**)). Each value represents the mean ± SD (*n* = 6–8). Different letters indicate a significant difference (*p* < 0.05) based on one-way ANOVA and Tukey’s HSD test (in (**B**–**E**)).

In the following experiments, the interference of phenol red in the medium with the light transmission of different light sources was evaluated. The DMEM and RPMI media used in this study also contain 10 and 15 mg/mL of phenol red, respectively (Table A1), and their absorbance spectra showed strong absorption in the 550–560 nm region of the light. However, Pf-D did not exhibit any notable light-absorbance properties in the visible range (Figure 2A). After passing through 3 mL of RPMI and DMEM in a 3.5 mm culture dish, the light energy and illuminance were reduced to 88.6 and 80.4%, and 76.9 and 53.9%, respectively, when irradiated with FL at 5 W/m^2^ and 2000 lx (Figure 2B). The interference effect on the irradiated light increased as the volume of the medium increased, and both RPMI and DMEM showed the greatest impeding effect under the green LEDs (Figure 2C,D). In particular, the interference effect of DMEM on light transmission was greater than that of RPMI due to its higher phenol red content. The light energy of green light, white LEDs, FL, and blue LEDs decreased by 58, 28, 23, and 19%, respectively, after passing through a 3 mL volume of DMEM in a 3.5 mm dish (Figure 2D). When evaluating the change in light transmittance through the DMEM at different illuminances of the green and white LEDs, which were the most strongly interfered with by the medium, no significant differences were observed (Figure 2E).

The light-interfering effects of DMEM and RPMI media were greatest under the green LEDs (Figure 2C,D), which had the emission peak closest to the maximum absorbance of phenol red, while there was little light absorption for red light (Figure 2A). This suggests that phenol red plays the most important role in interfering with light transmission through the medium. In addition, Pf-D did not interfere with light transmission, suggesting that the effect of other non-coloring components in the medium is negligible. FL and white LEDs, on the other hand, have broad light emission peaks (Figure A1). This leads to light absorption by phenol red, and the resulting interference with light transmission appears to be relatively large.

### 2.3. Effect of Riboflavin and Phenol Red on Photosensitizing Properties of Culture Medium

**Figure 3:**
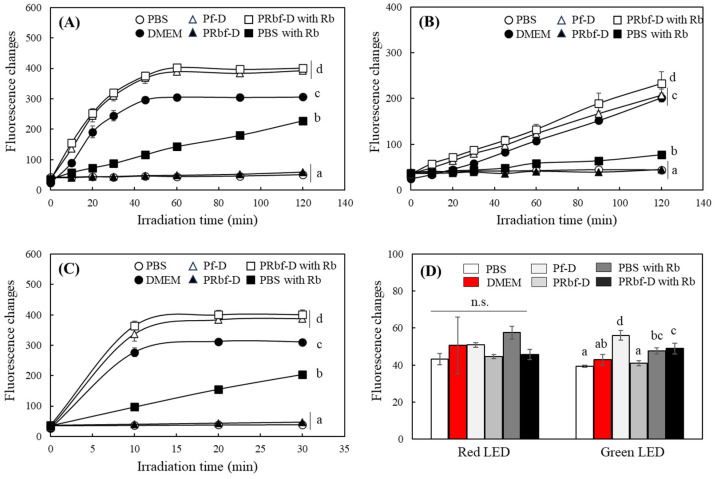
Changes in the photosensitizing properties of culture media by riboflavin and phenol red under different lights. Each medium, including regular DMEM, phenol red-free DMEM (Pf-D), phenol red- and riboflavin-free DMEM (PRbf-D), and PRbf-D or PBS supplemented with riboflavin 0.4 mg/L, was incubated under the irradiation of FL (2000 lx) (**A**), white (2000 lx) (**B**), blue (200 lx) (**C**), red, and green (each 2000 lx) (**D**) LEDs. Changes in the fluorescent intensity of DCFH were analyzed (ex. 485, em. 535 nm) at each time point. Each value represents the mean ± SD (*n* = 3). Different letters indicate a significant difference (*p* < 0.05) based on one-way ANOVA and Tukey’s HSD test. n.s.; not significant.

The role of riboflavin and phenol red in presenting the photosensitizing properties of culture media was evaluated using the media with or without riboflavin and phenol red. The increases in the DCF fluorescence of PBS containing 0.4 mg/mL of riboflavin, DMEM, Pf-D, and PRbf-D supplemented with 0.4 mg/mL of riboflavin were 228.2, 306.0, 393.3, and 401.5, respectively, after FL irradiation for 120 min; no significant change was observed in PBS and PRbf-D (Figure 3A). A similar order of results was observed with exposure to white or blue LEDs; the increase in the photosensitizing property, as represented by the increase in DCF fluorescence, was maximized in riboflavin-containing and phenol red-free media (Figure 3B,C). When irradiated with red or green LEDs, all media showed very weak photosensitizing properties, similar to the results shown in Figure 1. Irradiation with red LEDs did not cause a significant difference in the photosensitizing activity of the media, regardless of the presence of riboflavin or phenol red. However, there were some significant differences in Pf-D- and riboflavin-containing media under green LEDs (Figure 3D). Phenol red also significantly affected the expression of the photosensitizing property of the medium. However, the increase in DCF fluorescence by the addition of riboflavin and irradiation with blue light appropriate for the photosensitization of riboflavin was particularly large and rapid.

It is noteworthy that the addition of riboflavin to PBS induced a significantly lower increase in DCF fluorescence than when riboflavin was added to the medium. The previous results indicate that ROS production by photosensitization of riboflavin under UV is significantly enhanced in the presence of vitamins or amino acids such as tryptophan, tyrosine, and folic acid [27]. Cell culture media such as DMEM, unlike PBS, contain a wide variety of nutrients such as amino acids, vitamins, and minerals. Therefore, interactions with various medium components are likely to enhance the production of ROS by riboflavin photosensitization.

The fluorescence reaction of 2′,7′-dichlorofluorescin (DCFH) with ROS is a widely used for detecting and quantifying oxidative stress in biological systems. DCFH is susceptible to oxidation into DCF by various ROS, including superoxide radicals, hydroxyl radicals, and peroxynitrite, and emits fluorescence [28]. We have taken advantage of this property of DCFH to successfully measure the photosensitizing activity of each medium based on the ROS levels generated upon exposure to light. This is considered a novel approach that has rarely been reported on in previous studies and needs to be established as a useful method for quantifying photosensitizing activity in future studies.

### 2.4. Phototoxicity of HaCaT in Different Cell Culture Media

HaCaT cells are an immortalized human keratinocyte cell line derived from adult human skin. They are widely used in skin biology cellular research and in light irradiation studies [29]. HaCaT cells are typically maintained in standard cell culture conditions using DMEM with 5–10% fetal bovine serum (FBS). In the following experiments, HaCaT cells were irradiated with light in different medium conditions, and the resulting changes in cell viability were evaluated.

**Figure 4:**
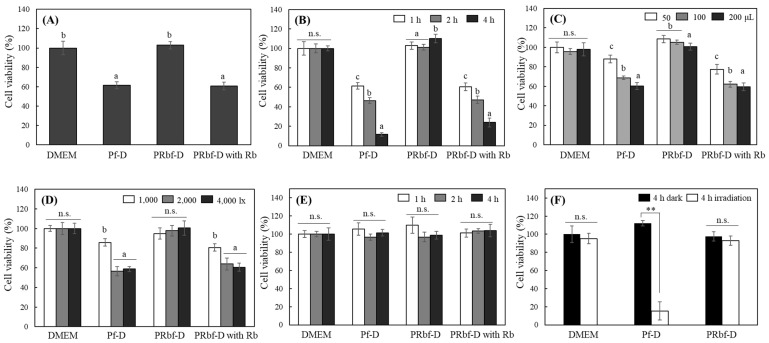
Modulation of HaCaT viability by different culture conditions under fluorescent light. HaCaT was incubated in different media (200 μL) for 1 h (**A**), for different irradiation times (1–4 h) (**B**), in different volumes (50, 100, and 200 μL) of medium for 1 h (**C**) under the irradiation of FL (2000 lx), and with different intensities (1000–4000 lx) of the irradiation for 1 h (**D**). Each irradiated medium was then replaced with regular-growth DMEM, and the cells were further grown for 18 h. Cell viability was analyzed using the MTT assay. HaCaT viability in the dark (**E**) and the viability difference between cells stored in the dark and irradiated for 4 h (**F**) were also analyzed. Each value represents the mean ± SD (*n* = 6–8). Different letters indicate a significant difference (*p* < 0.05) based on one-way ANOVA and Tukey’s HSD test. Significantly different from each other based on Student’s *t*-test (**, *p* < 0.01 in (**F**)). n.s.; not significant.

When cultured HaCaT cells were exposed to 2000 lx of FL for 1 h, significant differences in phototoxicity were observed according to the presence of phenol red and riboflavin in the media (Figure 4A). Phototoxicity was significantly enhanced in cells with Pf-D compared to those with regular DMEM. While cells in PRbf-D showed little phototoxicity, the addition of riboflavin to PRbf-D again significantly reduced cell viability. When the light exposure time was extended from 1 to 4 h, no difference in phototoxicity was observed in cells with DMEM and PRbf-D, but cell viability decreased with increasing FL irradiation time in Pf-D and PRbf-D supplemented with riboflavin (Figure 4B). When different media volumes were applied, cells with DMEM showed no difference in phototoxicity based on the amount of media treated, while some changes in PRbf-D were observed. However, phototoxicity was enhanced as the volume of treatment medium increased in cell with Pf-D and PRbf-D with riboflavin (Figure 4C). This result is likely due to the increase in the absolute amount of photosensitizable riboflavin in the medium. When cells were irradiated with FL with different light intensities ranging from 1000–4000 lx, no changes in cell viability were observed in cells irradiated in DMEM and PRbf-D media, but a significant increase in cytotoxicity was observed between 1000 and 2000 lx in Pf-D and PRbf-D with riboflavin. When the light intensity was enhanced to 4000 lx, there was no significant difference from 2000 lx (Figure 4D); this is likely due to the photosensitization of all riboflavin in the medium at 2000 lx. Cell viability was not affected by different media or changes in incubation time when cells were stored in the dark (Figure 4E).

Interestingly, the viability of HaCaT cells irradiated with FL for 4 h in DMEM showed little difference from that of cells kept in the dark. Only cells irradiated with Pf-D showed significant phototoxicity (Figure 4F). Although DMEM possessed photosensitizing properties due to its riboflavin content, cells irradiated with the medium showed negligible activity in inducing phototoxicity. Of the media used in this study, only DMEM contained an excess amount of sodium pyruvate (Table A1). Sodium pyruvate can directly react with H_2_O_2_ in a non-enzymatic reaction and converts H_2_O_2_ into water and CO_2_, effectively neutralizing its oxidative potential. Accordingly, sodium pyruvate is often added to cell culture media as a protective agent to scavenge ROS that may be generated during cell metabolism or due to external stressors. Therefore, in the following experiments, the relationship between the level of H_2_O_2_ produced in the medium under light irradiation and cytotoxicity was investigated.

### 2.5. H_2_O_2_ Levels in Different Culture Media and Its Involvement of Phototoxicity

**Figure 5:**
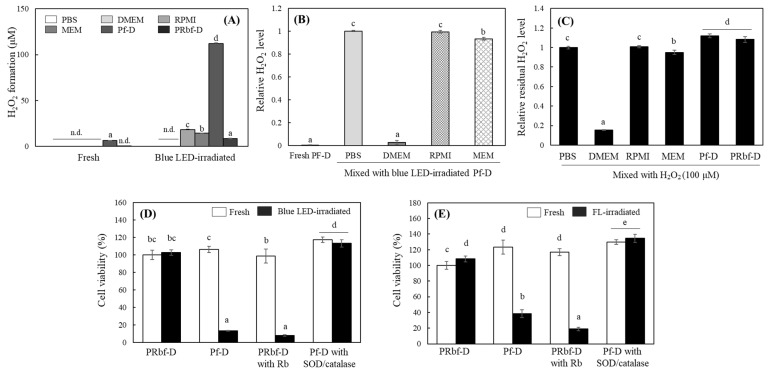
Changes of H_2_O_2_ levels in different media by light irradiation and effects on HaCaT cytotoxicity. Each medium was exposed to blue LEDs (200 lx, 1 h) under cell culture conditions, and H_2_O_2_ levels formed in fresh or the blue LED-irradiated media were measured (**A**). The blue LED-irradiated Pf-D was mixed with different fresh media and H_2_O_2_ levels in the mixture were analyzed after 30 min incubation (**B**). H_2_O_2_ (100 M) was added to different media and the residual levels were also analyzed after 1 h incubation (**C**). HaCaT were grown in a regular DMEM for 20 h and were replaced with 200 μL of fresh, blue LED-irradiated (200 lx, 1 h) (**D**) or FL-irradiated medium (2000 lx, 1 h) (**E**) in the absence or presence of SOD/catalase. The cells were further incubated in the dark for 24 h. Each value represents the mean ± SD (*n* = 3 in A-C or *n* = 8 in (**D**,**E**)). Different letters indicate a significant difference (*p* < 0.05) based on one-way ANOVA and Tukey’s HSD test. n.d.; not detected.

Since photosensitizers can generate ROS directly or indirectly by receiving light, it was expected that ROS would be importantly involved in the cytotoxicity induced in the light-irradiated cell culture medium. Therefore, different media were irradiated with blue LEDs, and the amount of H_2_O_2_ generated in the media was quantified using the ferrous oxidation-xylenol orange (FOX) assay (Figure 5). In fresh unirradiated medium, H_2_O_2_ was not detected except at negligible levels in Pf-D. Under blue LED radiation, the amount of H_2_O_2_ in PBS and DMEM was below the detection level. However, the H_2_O_2_ levels generated in RPMI and MEM were detected at 18.4 and 14.5 μM, respectively, after 1 h of blue LED irradiation (200 lx). However, 112.1 μM of H_2_O_2_ was generated in Pf-D, which was attributed to the absence of both sodium pyruvate and phenol red in Pf-D, eliminating the quenching effect of H_2_O_2_ and interference with light, respectively (Figure 5A).

To determine whether H_2_O_2_ produced in the medium by light irradiation is removed by medium components, such as sodium pyruvate, the residual amount of H_2_O_2_ was measured after a 1:1 mixing of different fresh medium with Pf-D irradiated with blue light to produce H_2_O_2_. H_2_O_2_ in Pf-D was slightly reduced by mixing with MEM, but in particular, it was almost eliminated by mixing with DMEM (Figure 5B). Direct addition of H_2_O_2_ to each medium resulted in a decrease and increase of H_2_O_2_ within 10% in MEM or Pf-D and PRbf-D, whereas more than 80% was removed in DMEM. These results indicate that certain medium components present only in DMEM were primarily involved in quenching H_2_O_2_; the DMEM used in this study contained 110 μg/L sodium pyruvate (Table A1).

Therefore, the phototoxicity to HaCaT shown in the results in Figure 4 is probably due to ROS, including H_2_O_2_, generated in the medium by light. In the following experiments, HaCaT cells were treated with media pre-irradiated with blue LEDs or FL, and cell viability was compared to cells treated with fresh media. No difference in cell viability was observed in media treated with PRbf-D regardless of light irradiation, but cells treated with Pf-D or PRbf-D media containing riboflavin pre-irradiated with 200 lx blue LEDs were almost destroyed (Figure 5D). Similar results were observed with FL-irradiated media, with a significant reduction in cell viability of HaCaT treated with Pf-D and PRbf-D containing riboflavin irradiated with 2000 lx FL (Figure 5E). Interestingly, the addition of superoxide dismutase (SOD)/catalase to media irradiated with either blue LEDs or FL completely restored cell viability. These results suggest that the removal of ROS by the action of sodium pyruvate or SOD/catalase can neutralize the phototoxicity of the medium, and thus ROS generated in the medium by light irradiation are the main cause of the induction of cell death.

### 2.6. Effects of Serum on ROS Levels in Media

**Figure 6:**
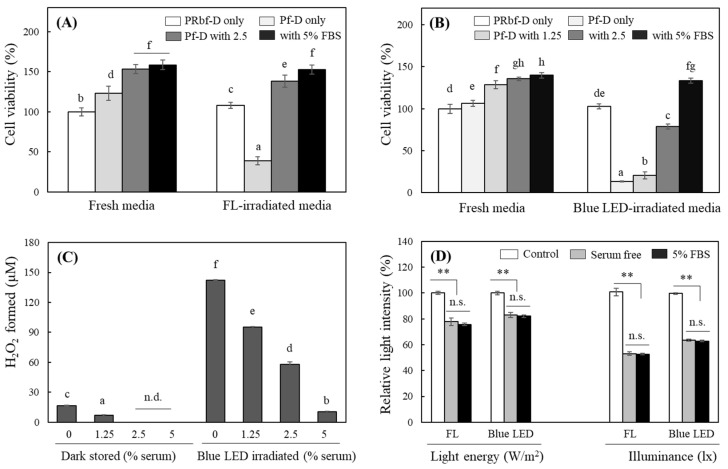
Effects of FBS on cytotoxic property, H_2_O_2_ levels of light-irradiated media and light transmittance. HaCaT were grown in a regular DMEM and were replaced with fresh, FL-irradiated (2000 lx, 1 h) (**A**) or blue LED-irradiated (200 lx, 1 h) PRbf-D or Pf-D (**B**) in the presence of different serum contents. The cells were further incubated in the dark for 24 h, and cell viability was analyzed using the MTT assay. Pf-D containing different serum contents was exposed in the dark or under the irradiation of blue LEDs (200 lx, 1 h) under cell culture conditions, and the H_2_O_2_ levels formed in each medium were measured (**C**). Changes of light transmittance of FL (5 W/m^2^ or 2000 lx) and blue LEDs (5 W/m^2^ or 100 lx) through the DMEM (3 mL) containing serum were also analyzed (**D**). Each value represents the mean ± SD (*n* = 8). Different letters indicate a significant difference (*p* < 0.05) based on one-way ANOVA and Tukey’s HSD test. ** Significantly different from each other based on Student’s *t*-test (*p* < 0.01, in (**D**)).

FBS is a common supplement added to cell culture media for growing and maintaining cells. Since FBS contains a wide range of serum substances including growth factors, hormones, proteins, vitamins, and minerals, it was assumed that these would affect the light sensitivity of the medium or the changes in the medium upon exposure to light. To evaluate the effect of serum, media containing different contents of FBS irradiated with FL or blue LEDs were treated with HaCaT cells for 24 h. The results showed that cell growth was stimulated with increasing concentrations of FBS in the medium in the case of non-light irradiation. Cells cultured in Pf-D pre-irradiated with 2000 lx FL showed a decrease in viability of more than 60%, whereas cells treated with the same light-irradiated Pf-D containing 2.5 and 5% FBS showed a complete recovery of cytotoxicity and actually promoted cell growth (Figure 6A). Irradiation of Pf-D with 200 lx blue LEDs resulted in stronger cytotoxicity than FL but still showed an FBS concentration-dependent recovery of cytotoxicity, and rather, an increase in cell viability was observed in the medium containing 5% FBS (Figure 6B).

As described above, FBS contains a variety of serum components, some of which are expected to scavenge ROS, which are responsible for cytotoxicity in light-exposed media. Pf-D medium containing different content of FBS was irradiated with blue LEDs for 1 h under cell culture conditions, and the level of H_2_O_2_ in the medium was analyzed. Blue LED exposure induced the generation of high levels of H_2_O_2_, which decreased with the concentration of FBS in the medium (Figure 6C). The interference of FBS in FL or blue LED transmission in DMEM was evaluated. It was found that light transmission through the medium containing 5% FBS resulted in a 1–2% decrease in energy or illuminance, which was not significantly different from serum-free media (Figure 6D). These results suggest that the FBS in the medium interferes with ROS production under light irradiation because it contains many components with antioxidant potential [30]; it does not interfere with the transmission of light within the visible light spectrum.

### 2.7. Effects of Culture Media on the Behavior of Added Photosensitizers

Photosensitizers receiving light cause their own chemical changes or transfer energy to oxygen or surrounding molecules to generate ROS directly or indirectly. The ROS generated by photosensitizers can cause cytotoxicity by damaging biomolecules such as proteins, DNA, and lipids [31]. These properties of photosensitizers are currently utilized in various fields, such as the treatment of skin diseases (e.g., psoriasis), photodynamic therapy of various types of cancer, and inactivation of microorganisms.

**Figure 7:**
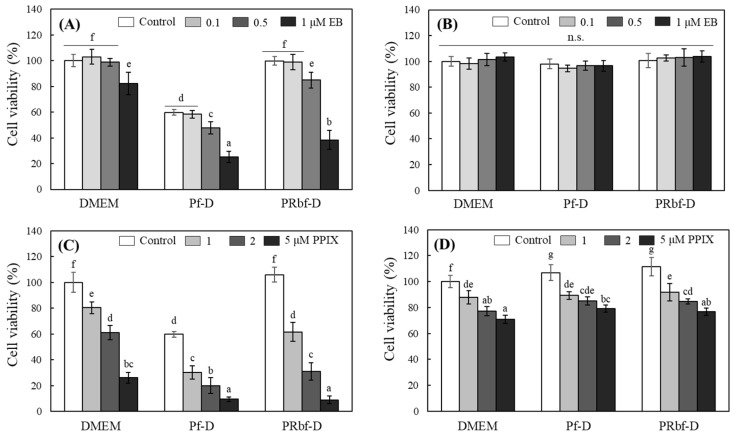
Effects of erythrosine B and protoporphyrin IX on HaCaT phototoxicity in different media. HaCaT was treated with EB (0–1 μM) (**A**,**B**) and PPIX (0–10 μM) (**C**,**D**) in different media, and the cells were exposed to FL (2000 lx) (**A**,**C**) or in the dark (**B**,**D**) for 1 h. The media were then replaced with regular complete DMEM, and the cells were further incubated for 18 h. The cell viability was measured by MTT assay. Each value represents the mean ± SD (*n* = 6–8). Different letters indicate a significant difference (*p* < 0.05) based on one-way ANOVA and Tukey’s HSD test. n.s.; not significant.

In the following experiments, changes in the cytotoxic behavior of photosensitizers such as erythrosine B (EB) or protoporphyrin IX (PPIX), which are used in photodynamic therapy, were investigated in different medium environments. Both EB and PPIX are commonly used as photosensitizers in photodynamic therapy. They generate ROS upon light exposure, leading to targeted cell damage, with applications ranging from antimicrobial treatments to potential cancer therapies [32,33]. HaCaT was treated with different cell culture medium containing EB or PPIX and then exposed to 2000 lx of FL for 1 h to induce phototoxicity. Significant cytotoxicity was induced by 1 h of FL irradiation in HaCaT treated with EB at a concentration of 1 μM in all DMEM, Pf-D, and PRbf-D media. The phototoxicity of EB by FL irradiation was apparently strongest in Pf-D, but it seemed to be strongly affected by riboflavin in the medium independent of EB. On the other hand, the phototoxicity of EB in regular DMEM was much less pronounced, probably due to the strong interfering effect of phenol red in the medium on EB, which has a similar maximum light absorption range of 500–550 nm. The phototoxicity of EB under FL irradiation in the PRbf-D medium in the absence of both phenol red and riboflavin was the most evident (Figure 7A). In the dark, HaCaT cell viability was not affected regardless of medium type or EB concentration (Figure 7B).

HaCaT was also treated with PPIX, a porphyrin-based photosensitizer, in each medium and exposed to 2000 lx of FL. Phototoxicity induced by PPIX was dependent on its concentration in all the treated media, with the lowest cell viability observed in the Pf-D. However, PPIX showed the most pronounced difference in concentration-dependent phototoxicity in the PRbf-D medium, where there was no interference from riboflavin and phenol red (Figure 7C). It is interesting to note that PPIX was shown to reduce cell viability in the dark, regardless of the culture medium being treated, but to a lesser extent than FL irradiation (Figure 7D). It was reported that porphyrin-derived photosensitizers induced decolorization of formazan independent of cell viability when cell viability was assessed by 3-(4,5-dimethylthiazol-2-yl)-2,5-diphenyltetrazoliumbromide (MTT) method [34]. The present results are consistent with this previous report, and, therefore, the PPIX-induced changes in MTT responses in the dark do not appear to be related to the culture media.

The present results show that phenol red, a pH indicator in the medium, can severely interfere with light transmission. In particular, it can reduce the activity of photosensitizers with similar maximum light absorption wavelengths, such as EB. In addition, riboflavin in the medium can offset the activity of various photosensitizers, and medium components such as FBS and sodium pyruvate can interfere with related experiments by scavenging ROS generated by activation of photosensitizers. Therefore, for accurate measurement and understanding of photosensitivity in cell culture systems, it is recommended that media free of these components be used. The current results suggest that the presence of certain factors in commonly used media can cause a significant effect and should be considered when evaluating the light-derived effects of compounds with different photosensitizing activities and the mechanisms involved in cell culture systems.

## 3. Materials and Methods

### 3.1. Chemicals and Culture Media

Riboflavin, PPIX, EB, SOD, catalase, xylenol orange, and 2′-7′dichlorofluorescin diacetate (DCFH-DA) were purchased from Sigma-Aldrich chemical Co. (St. Louis, MO, USA). MTT was from Amresco Inc. (Solon, OH, USA). Riboflavin (10 mM), PPIX (10 mM), and EB (50 mM) dissolved in dimethyl sulfoxide (DMSO) were aliquoted and stored at −80 °C. DMEM (LM 001-05), Pf-D (LM 001-10), PRbf-D (LM 001-234), RPMI (LM 011-01), MEM (LM 007-07), and FBS were purchased from Welgene (Gyeongsan, Republic of Korea). Table A1 shows the main components of the media used in the experiment.

### 3.2. Cell Line and Maintenance

HaCaT, an immortalized human keratinocyte, was obtained from Amore Pacific Corporation (Seoul, Republic of Korea). The cells were maintained in DMEM and supplemented with 10% FBS, 100 unit/mL penicillin, and 0.1 mg/mL streptomycin. The growth condition of the cells was set to 37 °C at 95% humidity and 5% CO_2_.

### 3.3. Measurement of Photosensitizing Property

The photosensitizing properties of each culture medium were evaluated under irradiation with red, green, blue, and white LEDs (Bissol LED Inc., Seoul, Republic of Korea) and FL (FPL 27 W, 9000 K, Cosmos Electric Co., Seoul, Republic of Korea) using a DCFH probe. The profiles of the emission spectra of the light sources used in this study are shown in Figure A1. DCFH was prepared by incubating DCFH-DA (25 μM) with HCT 116 cells for 2 h at 37 °C in the dark to remove diacetate in the cells. After the cells were washed with ice-cold PBS and lysed using DMSO, the cell lysates were aliquoted and stored at −80 °C and diluted in distilled water (DW) before use. The protein concentration of the diluted cell lysates containing DCFH was determined to be 133.7 μg/mL using the BCA assay. A mixture of the DCFH solution (50 μL) with each medium (50 μL) was incubated under FL (2000 lx), different LEDs (blue 200, green 2000, red 2000, and white 2000 lx), or in the dark at room temperature. At different time points, changes in DCF fluorescence were analyzed at excitation/emission 485/535 nm (cut off 530 nm) (Spectra Max M2, Molecular device, Sunnyvale, CA, USA).

### 3.4. Analysis of Light Transmittance Changes by Medium

Cell culture dishes (35 mm × 10 mm, #430165 polystyrene, Corning Inc., Corning, NY, USA) containing different volumes of each medium (0–3 mL) were exposed under different lights including FL and white, blue, green, and red LEDs. The intensity of light irradiation was set to 5 W/m^2^ for energy and 2000 lx for illuminance (except for l00 lx of the blue LEDs) based on the transmittance of the empty dish. The light energy (W/m^2^) and illuminance (lx) after transmission of the dishes containing different media was analyzed using an irradiance meter (IRR1-Sol, Fluke, Everett, WA, USA) and a light meter (Traceable 62344-944, VWR, Radnor, PA, USA) respectively. The transmittance of green and white LEDs passed through a dish containing 3 mL of DMEM was also analyzed at different illuminances (0–2000 and 0–4000, respectively).

### 3.5. Evaluation of Cytotoxic Properties

The cytotoxic and phototoxic effects on HaCaT were determined using the MTT assay. The cells were seeded in 96-well plates at 1.0×10^4^ cells per well and grown for 20 h at 37 °C. The growth media were replaced with different volumes (50–200 μL) of media and irradiated with FL at a light intensity of 1000–4000 lx for 0–4 h. In order to evaluate phototoxic effects of photosensitizers, the cells were irradiated in different media containing EB or PPIX under FL (2000 lx) for 1 h. The irradiated media or the media containing EB or PPIX were then replaced with complete DMEM, and the cells were allowed to grow further for 18 h. In another experiment, each medium was pre-irradiated with blue LEDs 200 lx or FL 2000 lx for 1 h, and the grown cells were treated 200 μL of the irradiated medium for 24 h. The medium of the treated cells was removed and replaced with a fresh medium containing 0.5 mg/mL MTT, and the cells were further incubated at 37 °C for 1 h. After solubilizing MTT formazan in cells with 100 µL of DMSO, the absorbance was measured at 550 nm (Spectra Max M2).

### 3.6. Measurement of H_2_O_2_ Levels

H_2_O_2_ levels in different cell culture media were analyzed using the previous method based on the FOX assay [35]. The working solution for the FOX assay was prepared by adding 1 mM ammonium ferrous sulfate to a mixture (1:1, *v*/*v*) of 400 μM xylenol orange (in DW) and 800 mM D-sorbitol (in 200 mM H_2_SO_4_). Each 3 mL of medium was prepared in a 6-well plate and was irradiated under blue LEDs (200 lx) for 1 h under cell culture conditions (5% CO_2_, 95% humidity, 37 °C). The irradiated or fresh medium (40 μL) was reacted with FOX working solution (160 μL) for 45 min in a dark place, and absorbance was measured at 550 nm. In another experiment, the irradiated Pf-D medium (20 μL) or 100 μM H_2_O_2_ (20 μL) was mixed with different fresh media (20 μL), and the mixture was incubated for 30 min in the dark. Then residual levels of H_2_O_2_ in each sample were analyzed.

### 3.7. Data Analysis

All values represent the mean ± standard deviation (SD). Each experiment was repeated 3–8 times. Statistical significance was evaluated using Student’s *t*-test. One-way ANOVA with Tukey’s HSD (honestly significant difference) test (*p* < 0.05) was also used for comparing multiple results using the SPSS program (IBM SPSS Statistics 24, SPSS Inc. Chicago, IL, USA).

## 4. Conclusions

In the present study, the photosensitizing property of cell culture medium and the consequent effect on HaCaT keratinocytes upon light exposure were investigated. Commonly used cell culture media, namely DMEM, RPMI, and MEM, showed significant photosensitizing responses leading to the generation of ROS when exposed to different light sources. Riboflavin content was the main determinant of the potency of the photosensitizing property of the media. The pH indicator dye phenol red also significantly impeded light transmission and reduced the photosensitivity of the medium. In media without riboflavin and phenol red, phototoxicity to HaCaT was maximized. The ROS generated under light irradiation played a critical role in inducing phototoxicity in cells. The presence of sodium pyruvate or serum, however, significantly reduced phototoxicity by quenching H_2_O_2_ in the media. In conclusion, the results of this study suggest that cell culture media can exhibit photosensitizing properties mainly due to riboflavin, and several medium components including phenol red, sodium pyruvate, and FBS could be confounding factors by interfering with light transmission and by quenching ROS generated by light irradiation, which should be carefully considered in experiments related to light exposure of cells.

## Data Availability

Data available from the corresponding author upon reasonable request.

## Appendix A

**Table A1:** Major constituents of the cell culture media used in the present study.

	RPMI	MEM	DMEM	Rf-D	PRbf-D
Cat. No.	LM 011-01	LM 007-07	LM 001-05	LM 001-10	LM 001-234
Phenol red (mg/L)	5	10	15	-	-
Sodium pyruvate (mg/L)	-	-	110	-	-
Riboflavin (mg/L)	0.2	0.1	0.4	0.4	-
